# Clinicopathological pattern of breast lesions in children and adolescents

**DOI:** 10.4314/ahs.v23i3.28

**Published:** 2023-09

**Authors:** Chukwuemeka Charles Nwafor, Kingsley Uwaemechi Umeh, Emmanuel Benjamin Etuk, Uchechukwu Brian Eziagu, Ikwo Jonathan Kudamnya, Esther Ekwo

**Affiliations:** 1 Department of Pathology, University of Uyo; 2 Department of Surgery, University of Uyo; 3 Department of Histopathology, University of Uyo Teaching Hospital

**Keywords:** Adolescents, benign, breast lesions, fibroadenoma

## Abstract

**Background:**

Breast lesions are not common in children and adolescents. The aim of this study is to retrospectively survey the clinicopathological pattern of breast lesions in children and adolescents in our setting.

**Materials and method:**

This is a retrospective study of all breast specimens from children and adolescents that were histopathologically diagnosed in University of Uyo Teaching Hospital.

**Results:**

The youngest patients seen were 11 years old, with mean age of 17.1 ± 1.91. The commonest clinical diagnoses were fibroadenoma (n=134, 72.4%). Thirty-five-point seven percent of the patients presented within 6 months of noticing the lump. The mean size of the lumps was 6.2cm ± 3.9. Fibroadenoma was the most common benign diagnosis and the most common histopathologic diagnosis in this study. The mean age of patients with fibroadenoma was 17.15±1.83.

**Conclusion:**

The pattern of breast lesions in adolescents in Uyo is similar to that from other parts of Nigeria.

## Introduction

Children and adolescents are a special group of people between ages 0-19 years. Children are individuals aged between 0-9 years, while adolescents are individuals aged between 10 and 19 years. Adolescents make up about 16% of the world's population.^1^ The mammary gland of the human breast is characterized by cellular pliability (potential for transformation), with extensive remodelling through puberty and adulthood. This key characteristic feature of the mammary gland, unfortunately increases its susceptibility to malignant transformation (carcinogenesis).2 Yearly about 400,000 children and adolescents are diagnosed of cancers, which include mainly the so-called childhood tumors (leukaemias, lymphomas, brain tumors, neuroblastomas and nephroblastomas).^1^,^3^-^4^ Globally childhood cancer is the sixth leading cause of total cancer burden and the ninth leading cause of childhood disease.^5^ Approximately 1 in 285 children will be diagnosed with cancer before age 20 years.^6^ All spectrum of breast lesions seen in adults can be seen in children and adolescents, though breast cancers are said to be extremely rare in people aged 19 years and below. Most childhood and adolescent neoplastic breast lesions are benign, of which fibroadenomas are the commonest accounting for 80-95% of these lesions. The less common benign breast neoplasms in this age group, are fibrocystic change, hamartomas and phyllodes tumor. Malignant breast lesions account for 0.02% of breast lumps in individuals <19 years of age and are mostly metastatic breast lesions. The primary sources of these metastatic lesions could be rhabdomyosarcomas, lymphomas or neuroblastomas.^7^-^19^

Interestingly, children and adolescents with breast pathologies form a significant proportion, of patients seen in surgical outpatient clinics of University of Uyo Teaching Hospital (UUTH) and to the best of our knowledge, the clinicopathological pattern of these breast specimens have not been documented. The documentation, comparison with local and international studies and relevant literature review is what this paper aims to address.

## Materials and method

This is a retrospective study of all breast specimens from children and adolescents that were histopathologically diagnosed in the Histopathology department of UUTH over a 13 year period from January 1, 2008 to December 31, 2020. This histopathology laboratory is the main facility where histopathology services are rendered in Akwa Ibom State and as such render's services to the host hospital, secondary health facilities and many privately-owned hospitals within the State. All surgeries were handled as day cases, using local anesthesia. Incisions used were circumareolar or curvilinear. The breast specimens received included excision biopsies, incision biopsies and core needle biopsies. These breast specimens were received in 10% buffered formalin and auto processed. The paraffin embedded sections (sectioned at 2–3 µm) were routinely stained with hematoxylin and eosin (H and E) stains. Data were extracted from the departmental registers, patient request forms, duplicate copies of histopathology reports of all cases and case notes/files of patients. Information extracted include age, sex, duration of symptom before the presentation, laterality of breast lump, the maximum diameter of a breast lump, the clinical diagnosis made by the unit consultant or requesting physician, type of biopsy done and histopathologic diagnosis. The tumors were classified using the 2012 WHO International Classification of breast tumors.^20^ The data thus generated were analysed using statistical package for social sciences (SPSS) version 20 (IBM, SPSS Inc., Chicago, IL, USA). Simple frequencies were determined for categorical variables and mean was evaluated for continuous data. Chi square tests were used to determine significant difference of categorical variables. A p value of <0.05 was considered significant. All reports with ambiguous histological conclusions were excluded from the study. Four cases were excluded on account of missing/inadequate identification parameters (such as sex, age, and diagnosis).

## Results

**Figure 1:**
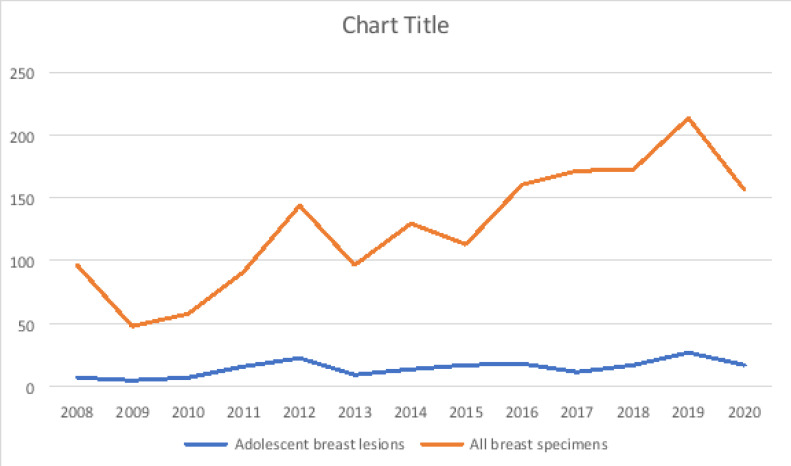
Yearly distribution of breast specimens in the adolescents, in relation to all breast specimens

A total of 185 breast specimens were from adolescents, accounting for 11.2% of all breast specimens (n=1646) seen during the period of study. Compared to the total number of breast specimens received, the volume of breast specimens from adolescents was fairly uniform except for two spikes in year 2012 and 2019 as shown in figure 1.

No breast specimen was from a child. The youngest patients seen were 11 years old, with a mean age of 17.1 ± 1.91. They patients were all females. There was an increase in the number of cases, with increase in age of the patients, except for mild drop noticed at ages 15 and 19 years, as shown in table 1.

**Table 1:** Age distribution of cases

Age	Frequency	Percentage
11	3	1.6
12	3	1.6
13	5	2.7
14	11	5.9
15	10	5.4
16	25	13.5
17	25	13.5
18	56	30.3
19	47	25.4
Total	185	100

Various parameters in relation to the breast specimens are shown in table 2. The commonest clinical diagnoses were fibroadenoma (n=134, 72.4%), while breast cancers were suspected in 1.1% (n=2) of cases. In 13 (7%) cases, phylloides tumor was the clinical diagnosis. Forty-two-point seven percent of the specimens were from the right breast, 36.2% from the left breast and 13.5% from both breasts. Breast lump was the presenting complaint in all the cases, while in 14 cases (7.6%), the lump was painful. Six (3.2%) patients, had single ipsilateral axillary lymph node enlargement. Thirty-five-point seven percent, of the patients presented within 6 months of noticing the lump, while 2.7% (n=5) presented after 60 months (5 years). Thirty-five-point seven percent of the specimens were greater than 2cm, but less than 5cm in their maximum diameter, while 27% of them, were greater than 5cm but less than 10cm in the widest diameter. The smallest specimen was 1cm and the biggest 22cm, with a mean size of 6.2cm ± 3.9. The commonest surgical procedure performed was excision biopsy (88.1%) and this is distantly followed by trucut biopsies (5.9%).

**Table 2:** Various characteristics of the breast lump

Various parameters	Frequency	Percentage (%)
Clinical diagnosis		
Fibroadenoma	134	72.4
Phylloides tumor	13	7
Breast cancer	2	1.1
Mastitis	1	0.5
Axillary breast	1	0.5
Benign breast disease	1	0.5
Not stated	33	17.8
Side (laterality)		
Right	79	42.7
Left	67	36.2
Both	25	13.5
Not stated	14	7.5
Duration before presentation		
< 6 months	66	35.7
7 – 12 months	15	8.1
13 – 24 months	38	20.6
25 – 59 months	7	3.8
60 months and above	5	2.7
Not stated	54	29.2
Surgical procedure		
Excision	173	93.5
Trucut	11	5.9
Incisional biopsy	1	0.5
Size		
< 2cm	20	10.8
2-5 cm	66	35.7
>5 <10 cm	50	27
> 10 cm	39	21.1
Not stated	10	5.4

The histopathologic diagnosis of all the lesions (98.9%) was of benign neoplastic nature except for a case of granulomatous mastitis (inflammatory) and a specimen that was not sufficient for histopathologic diagnosis as shown in table 3 and 4. Fibroadenoma was the most common benign diagnosis, as well as the most common histopathologic diagnosis in this study. The mean age of patients with fibroadenoma was 17.15±1.83 years. No malignant lesion (primary or secondary) was seen histopathologically, within the study population. There was a significant association between the clinical diagnosis and the histopathologic diagnoses, with a p value of 0.000. Of the 134 clinically diagnosed fibroadenoma, 116 (86.6%) were confirmed histopathologically as fibrodenoma (true positive). The remaining 18 cases, accounting for 9.7% (false positives), were rather diagnosed as various other benign neoplastic diagnosis [fibrocystic change (4.5%), tubular adenoma (3%), benign breast disease (3%), fibroadenomatoid hyperplasia (2.2%) and fibrolipoma (0.7%)]. The true negatives were 42 cases (22.7%) and the false negatives 9 cases (4.9%). One of the two (2) clinically diagnosed breast cancer cases was diagnosed histologically as fibroadenoma and the other one was not sufficient for histological diagnosis.

**Table 3:** Distribution of histopathologic diagnosis and mean ages

Histopathologic diagnoses	Frequency	Percentage	Mean age
Fibroadenoma	150	81.1	17.15 ± 1.83
Fibrocystic change	11	5.9	16.18 ± 2.86
Tubular adenoma	7	3.8	17.29 ± 1.50
Fibroadenomatoid hyperplasia	6	3.2	17.83 ± 1.12
Benign breast disease	6	3.2	17.00 ± 2.83
Others	5	2.5	
Total	185	100	

Others include: a case each of dermatofibroma, granulomatous mastitis, fibrolipoma, benign phylloides and not sufficient for histologic diagnosis.

**Table 4:** Relationship of major clinical diagnosis to histologic diagnosis

			Histologic diagnosis						
Clinical diagnosis	Fibroadenoma	Fibrocystic change	Tubular adenoma	Benign breast disease	Fibroadenomatoid hyperplasia	Fibrolipoma	Benign phylloides	NSD	Total
Fibroadenoma	116	6	4	4	3	1	-	-	134
Phylloides tumor	7	2	1	1	-	-	1	-	13
Breast cancer	1	-	-	-	-	-	-	1	2

NSD: Not sufficient for histologic diagnosis

Table 5, summarizes the relationship between diagnostic accuracy of clinical examination (clinical diagnosis) and histopathologic diagnosis, with a sensitivity of 92.8%, specificity of 70% and a positive predictive value of 86.6%.

**Table 5:** Diagnostic accuracy of clinical examination in UUTH

Diagnostic modality	Sensitivity	Specificity	Positive predictive value
Clinical examination %	92.8%	70%	86.6%

## Discussion

The presence of any breast swelling or mass lesions in young individuals usually causes great worry and anxiety to their parents, care givers and older siblings, due to their awareness/knowledge of morbidity and mortality associated with breast cancer in the population as well as the media hype about it.^10^,^11^ Studies have shown that breast lumps in children and adolescents are common in blacks.^21^ Breast masses involving adolescents in this study agreed with that and accounted for 11.2% of all breast specimens seen over 13 years. This rate is similar to 11.1% and 11.5% reported in Benin from 2 different studies and 11.5% reported in Enugu, Eastern Nigeria.^9^,^10^,^12^ The index rate is less than 16% observed in Abuja.^8^ All these Nigerian rates are much higher than an average rate of 3.2% reported in literature from similar western studies. Also, breast lumps are said to occur 2 years earlier in black teenagers.^14^,^21^,^23^

Studies have shown breast masses in children are uncommon, rising steadily with age during the adolescent period to young adulthood.^24^ It is important to note that no child (below the age of 10 years) was seen in this study, while a steady increase in frequency of breast lumps were seen with increasing age, except for a mild decrease between ages 18 and 19 years. This pattern is similar to the findings in Abuja, North Central Nigeria.^8^ Further more similar studies by Olu-Eddo etal in Benin, Ozumba etal in Enugu and Ademuyiwa etal in Lagos all reported few cases in children (range of 0.3 – 2.5%), however they all still reported a steady increase in frequency with age. The index mean age of 17 years, is fairly similar to 16 years observed in Abuja, 16.9 years in Benin, 16 years in Lagos and 16 years in Vienna Austria.^8^,^9^,^11^,^13^ Apart from increase in incidence of breast masses with age, other possible reasons for the increase detection with age may be the association of teenage years with sexual awareness and hence this group of girls are able to notice changes in their bodies and make mention of it.^11^

None of our patients was a male, similar to findings by Ademuyiwa etal in Lagos, Nigeria and Tea etal in Vienna, Austria.^11^,^13^ Even those that reported male breast masses in children and adolescents documented about 1-3 cases only in their series. The female breast is prone to masses due to its larger volume, complex structure and subject to constant physiological changes, due to the influence hormones (especially during reproductive age).^25^,^26^

Adolescent breast lesions are said to slightly occur more on the right breast, though there is no known reason for this observation.^24^ In this study most of the lesions (42.7%) occurred on the right, which is similar to observations in Port Harcourt, Abuja, Lagos (all in Nigeria) and Bangalore in India.^8^,^11^,^18^,^27^ However Olu-Eddo etal in Benin, documented left breast predominance.^9^ Common in all reviewed studies is the location of the adolescent breast masses in the upper outer quadrat, due to the larger percentage of breast tissue in this quadrant, that dovetails towards the axillae.^11^,^24^

The greatest reason for the removal of childhood and adolescent breast masses/lumps is the fear of a malignant breast lesion, which is usually associated with severe morbidity and mortality. Tissue histopathology following such breast mass removal have been benign diagnoses in most instances, querying the reason for subjecting young breast to surgery by some surgeons, instead of using a conservative, non-operative approach (close monitoring/observation, ultra sound scan and fine needle aspiration cytology). The reasons for objecting to surgery include: occasional complete regression of clinical detectable breast mass in adolescents, rarity of malignancy in this age group and cosmetic disfigurement (such as asymmetry, hyperplasia and scarring). Other reasons are the belief that both biopsy and surgery may impair the development of the breast at puberty to full maturity, which may cause unilateral hypoplasia of the breast and with sequelae of defective breastfeeding in future if significant amount of breast glandular lobules are removed during such procedure. For these proponents of conservative, non-operative approach, surgeries should be individualized and offered in any of the following situations: where cosmesis is highly compromised (giant mass), bloody nipple discharge, persistent history of pain in the breast mass, rapid growth of the mass and when the patient has malignancies with predilection (metastasis) for the breasts.^11^,^13^,^28^-^30^ On the other side of the divide, are the surgeons that belief that all breast masses should be removed and histopathologically evaluated, because malignant breast lesions have been documented in children and adolescents, even in males. These malignant breast lesions can exist alone or could be an associated incidental finding within benign lesions like fibroadenoma.^8^-^10^,^12^,^13^,^31^,^32^ Also surgical removal, allays the anxiety of parents, patients and their relatives and has been proposed as the treatment of choice.^13^

Fibroadenomas were the commonest diagnosis in this study, similar to all other similar studies locally and globally reviewed for this study. The index fibroadenoma rate of 81.1% is similar to 82% in Port Harcourt and 80% in Bangalore India. The index rate is less than 86.8% in Lagos Nigeria and higher than 74.3% in Abuja Nigeria, 62.8% in Vienna Austria and 58.8% in Benin Nigeria.^8^,^9^,^11^,^13^,^18^,^27^ Clinically fibroadenomas are discrete, smooth, firm, non-tender, very mobile within the breast and not attached to any tissue. Occasionally, it could be multiple within the same breast. Histopathologically they appear as circumscribed masses, composed of distorted and compressed glands, surrounded by densely proliferated intralobular stroma.^33^

The commonality of fibroadenoma in surgical practice has made making an accurate clinical diagnosis easy with a sensitivity of 93.3%, with only history taking and thorough breast examination. This sensitivity, specificity and positive predictive value are increased if imaging studies (like ultra sound scan) and fine needle aspiration cytology are added.^28^,^34^ The index study sensitivity, specificity and positive predictive value are fairly similar to 93.3%, 58.8% and 85.7% respectively documented by Egwuonwu etal.^33^ How-ever the index values are less than sensitivity of 88.7%, specificity of 99.1% and positive predictive value of 98.5% documented by Eltahir etal.^35^ The reason for the obvious difference in specificity and positive predictive values are due to the fact that Eltahir etal did the triple test (clinical examination, breast imaging and FNAC), which were not routinely done in our centre.^35^ To further increase the reliability of our clinical diagnosis, there is need to include the other aspects of the triple assessment in our breast clinics.

Fibrocystic change characterized histopathologically by marked stromal fibrosis, adenosis and cystically dilated glands (some of which are lined by apocrine metaplastic epithelial cells) was the second most common histopathological diagnosis made in this study, which is similar to the finding in all previous local and international studies, except in Bangalore India, where phyllodes tumor was the second most common lesion. Though the authors did not give any reason for their observation, but we think that their small sample size of 15 cases only, may have contributed to this observation.^8^-^11^,^13^,^18^ The index rate of 5.9% is greater than 1.9% seen in Lagos, but less than 10.3% in Abuja, 16.4% in Benin, 17% in an earlier study in Benin and 9.3% in Austria.^8^-^10^,^13^

Seven percent of the clinical diagnosis in this study were phyllodes tumor. They were made based on clinical history of large, painless, rapidly growing breast masses. Histopathologically, only one (0.5%) was confirmed to be benign phyllodes while the remaining were confirmed to be fibroadenomas. Phyllodes tumor have been known to be difficult to differentiate from giant fibroadenomas clinically. 36 The index rate of 0.5% is less than the documented rate in all previous local and international studies, that gave a rate range of 2.2% - 20%.^8^-^10^,^12^,^13^,^18^ Phyllodes (also known as cystosarcoma phyllodes) tumor are unusual breast stromal lesions, that are uncommon in children and adolescents (less than 1%) but are mainly seen in women between ages 40-50 years. Cases of bilaterality have also been documented. Histopathologically, phyllodes tumors are similar to fibroadenomas but show more stromal proliferation. They are classified histopathologically into benign, intermediate or malignant types. The higher-grade tumors have increased mitotic rate and show similarity to sarcomas. Despite its histopathologic classification, all phyllodes tumors (benign, intermediate, and malignant) have potential to metastasize and recur locally.^13^,^33^,^36^-^38^

Just like the management of every other mass lesion in medicine, detailed patient history and physical examination are essential for evaluation of any breast mass. In relation to a breast mass, history of pain, family history of breast disease, nipple discharge, precipitating factors, change in mass size, duration and progression are essential in guiding diagnosis and management.^14^,^17^ Only 35.7% of the patients studied presented within 6 months of noticing the mass, while in Lagos, 61% of patients presented within 6 months of breast lump notice.^11^ In Nnewi, 37.8% presented within 12 months of noticing the breast lump.34 We agree with Ademuyiwa etal that the reason for the late presentation in our environment might be linked to our culture that may not allow the adolescents to be free about discussing issues about intimate parts of their body.^11^ The mean size of breast lumps seen in this study was 6.2cm which is greater than 3.6cm documented in Lagos.^11^

The major limitations of this study are inherent to its retrospective nature and the fact that it is a single centre data survey. Important histories like age at menarche, family history of breast cancer or history of contraceptive use were not known. Furthermore, imaging studies like ultrasound scan and FNAC were not routinely done.

## Conclusion

The incidence of benign breast lesions in adolescents in Uyo is high, similar to what is obtainable in other parts of Nigeria, while breast cancer is rare in this age group. Adolescent male breast lesions are very rare, while fibroadenomas are the most common breast pathology in adolescent females. This study will serve as a baseline data for future studies, that will aim at further characterization.
